# Isolation and Characterization of a Viable *Toxoplasma gondii* from Captive Caracal (*Caracal caracal*)

**DOI:** 10.3390/pathogens12121412

**Published:** 2023-11-30

**Authors:** Hongjie Ren, Gaohui Mao, Yan Zhang, Niuping Zhu, Qunchao Liang, Yibao Jiang, Yurong Yang

**Affiliations:** 1 College of Animal Science, Henan Agricultural University, Zhengzhou 450000, China; 2 College of Veterinary Medicine, Henan Agricultural University, Zhengzhou 450000, China; 3 Henan Yinji Jiabao Amusement Park Management Co., Ltd., Zhengzhou 452300, China

**Keywords:** *Toxoplasma gondii*, caracal (*Caracal caracal*), virulence, ToxoDB#5, transmission, China

## Abstract

*Toxoplasma gondii* is a widespread protozoan parasite that infects most warm-blooded animals, and felids can serve as intermediate and definitive hosts. Pathological diagnosis and serological and etiological investigations were conducted on a captive caracal (*Caracal caracal*) carcass collected from China in 2022. Pathological diagnosis revealed that cardiac insufficiency, pulmonary edema, hepatic failure, and renal insufficiency were the causes of the caracal’s death. A modified agglutination test (cut-off: 1:25) revealed that IgG antibodies against *T. gondii* were present in the myocardium juice (1:1600), ascitic fluid (1:3200), and hydropericardium (1:800). A viable *T. gondii* (TgCaracalCHn2) strain was isolated from the tissue samples (heart, brain, spleen, and skeletal muscle) of this caracal using a mouse bioassay. The genotype of TgCaracalCHn2 was ToxoDB#5 (Type II variant), as determined via RFLP-PCR. The strain was avirulent in Swiss mice and matched the prediction of ROP18 and ROP5 gene alleles of TgCaracalCHn2 (2/2). Mild tissue cysts (203 ± 265) were observed in mice brains after inoculation with TgCaracalCHn2 tachyzoites. ToxoDB#5 is the dominant genotype in North American wildlife, and this is the first documented isolation of *T. gondii* ToxoDB#5 from China. This indicates that caracal plays an important role in the transmission of this *T. gondii* genotype.

## 1. Introduction

*Toxoplasma gondii* is a successful obligate intracellular protozoan parasite that has infected various animals, including birds, humans, livestock, and marine mammals [1]. Felines are important in the epidemiology of *T. gondii* infection because they serve as the definitive hosts of *T. gondii* and can excrete millions of oocysts into the environment. Furthermore, cats can re-shed oocysts after secondary or tertiary *T. gondii* infections [2,3]. Under mild environmental conditions, infectious sporulated oocysts can survive for more than 1 year [1]. Moreover, felines do not show positive serum until 2–3 weeks after infection with *T. gondii*, but they would have completed the discharge of oocysts by this time [1]. Most felines are generally infected with *T. gondii* by eating undercooked meat containing *T. gondii* cysts or by ingesting water, vegetables, fruits, or soil contaminated with oocysts. The current positive rate of *T. gondii* among the wild felines worldwide is approximately 65% [4,5]. The seroprevalence of *T. gondii* in felines from China is 24% [4,6].

Caracal is a medium-sized felid and one of the most expansive extant carnivores that spread throughout Africa, Central Asia, and southwest Asia into India. Its population is declining in parts of Asia and Northern Africa, but it is common, and its population is stable in central and southern Africa (https://www.iucnredlist.org/, accessed on 10 September 2023). *Toxoplasma gondii* infection has been reported in caracals using serological evidence [7,8,9,10,11,12,13]. Furthermore, one viable *T. gondii* strain (TgCaracalCHn1, ToxoDB genotype #2) has been isolated from the striated muscles of a caracal, and *T. gondii* cysts have been observed in the leg and tongue muscles of another caracal [13]. These data indicate that the caracal could be an intermediate host for *T. gondii.* ToxoDB#5 is the main *T. gondii* genotype of wildlife species in North America [14,15,16,17,18], but little is known about this genotype in China.

The objective of the study was to screen *T. gondii* infection in caracal via pathological diagnosis and serological and etiological investigations. This will provide reference for evaluating the threat of *T. gondii* to public health and safety.

## 2. Materials and Methods

### 2.1. Sample Collection

On 8 June 2022, one captive caracal died in a zoo in Henan (34°46′ N, 113°39′ E), China. The carcass was transported to the Laboratory of Veterinary Pathology of Henan Agricultural University (Zhengzhou, China) for pathological diagnosis. Raw beef, whole chicken, and pork were fed to this caracal.

### 2.2. Detection of Antibodies against T. gondii in Tissue Juice of Caracal

The myocardium juice, hydroperitoneum, and hydropericardium were collected, diluted from 1:25 to 1:51,200, and tested for IgG antibodies against *T. gondii* using the modified agglutination test (MAT) [19]. The whole formalin-fixed *T. gondii* antigens were obtained from the University of Tennessee Research Foundation (Knoxville, TN, USA). The caracal was considered to have been exposed to *T. gondii* when the titer of antibodies against *T. gondii* was higher than 1:25. Reference sera were kindly supplied by J. P. Dubey (ARS, USDA). Simultaneously, serum samples from positive and negative controls were included in the same 96-well U plate.

### 2.3. Isolation of Viable T. gondii from Caracal Tissues Using Bioassay in Mice

Tissues of the heart, brain, spleen, and skeletal muscle (total weight of 50 g) from this caracal were homogenized and digested in pepsin solution [1]. The homogenates were injected subcutaneously into Swiss mice (n = 5) at 1 mL per mouse. The Laboratory Animal Center of Zhengzhou University (China) provided specific pathogen-free Swiss mice. The remaining homogenate was stored at −20 °C for molecular analysis. After injection, the illness, deaths, and clinical manifestations of mice were observed and documented daily. Tissue (brain and lung) smears of dead mice were examined for *T. gondii*. The serum of surviving mice was tested for *T. gondii* antibodies using a modified agglutination test (MAT) at 1:25 and 1:200 dilutions. If parasites were found in the lungs or brains of mice, the tissues (heart, brain, lungs, and spleen) of the mice were ground and subcutaneously passaged into new groups of mice (n = 5) to preserve the viable *T. gondii* strain.

### 2.4. Histopathological Analysis and Polymerase Chain Reaction (PCR) Amplification of T. gondii

Fresh caracal tissues, including brain, myocardium, liver, spleen, lungs, kidney, stomach, diaphragm, leg muscles, and intestines, were fixed in 10% (*v*/*v*) neutral buffered formalin, processed into conventional paraffin histological sections, and stained using hematoxylin and eosin (H&E) and immunohistochemistry (IHC). Rabbit anti-*T. gondii* serum was used as the primary antibody, and mouse anti-rabbit IgG conjugated with HRP/DAB was used as the secondary antibody (IHC detection kit, ab64264, Abcam, Waltham, MA, USA). The distribution of *T. gondii* parasites in caracal tissues was observed under a light microscope.

DNA was extracted from the caracal tissues (myocardium, liver, spleen, lungs, kidney, tongue, leg muscles, brain, lymph nodes, duodenum, jejunum, ileum, cecum, colon, rectum, pancreas, diaphragm, feces, and tissue pepsin digestion liquids) using a commercial DNA extraction kit (Tiangen Biotec Company, DP304, Beijing, China). PCR assays were performed to detect *T. gondii* using the specific primer pair TOX5/TOX8. The products of *T. gondii* were expected to be 450 bp in length [20].

### 2.5. Toxoplasma gondii Cell Cultivation and Genotyping

Tissue (brain, lungs, or mesenteric lymph nodes) homogenates of *T. gondii*-positive mice were seeded into Vero cells (RPMI 1640, 3% fetal bovine serum, 37 °C, and 5% CO_2_). DNA was extracted from *T. gondii* tachyzoites collected from the cell cultures. The *T. gondii* genotype was determined using PCR-RFLP with 10 genetic markers: SAG1, SAG2 (5′- and 3′-SAG2, alt. SAG2), SAG3, GRA6, BTUB, L358, PK1, c22-8, c29-2, and Apico [21]. *Toxoplasma gondii* virulence factors were identified by genotyping ROP5 and ROP18 polymorphisms [22]. *Toxoplasma gondii* reference DNA was included in all of the batches.

### 2.6. Evaluation of the Virulence of T. gondii Isolated from Caracal Using Swiss Mice

*Toxoplasma gondii* tachyzoites were collected from the cell culture and diluted 10-fold after counting with a blood count plate from 10^−1^ to 10^−7^ to reach an endpoint of <1 tachyzoite. Next, <1, 10^0^, 10^1^, 10^2^, 10^3^, 10^4^, 10^5^, and 10^6^ tachyzoites were inoculated intraperitoneally into five Swiss mice at each dilution. The clinical symptoms of the mice were observed and documented daily. The lungs, mesenteric lymph nodes, and brain impression smears of the dead mice were examined for *T. gondii*, and the tissues of the dead mice were fixed in 10% (*v*/*v*) neutral buffered formalin. At 30 DPI, serum samples from the surviving mice were analyzed for *T. gondii* antibodies using MAT, with titers of 1:25 and 1:200, and euthanized 60 DPI. Mouse brains were homogenized and diluted to 1 mL with saline (0.85% NaCl). Tissue cysts were counted in 50 µL of the homogenate in two fields of view, and the counting result was multiplied by 20 to acquire the number of tissue cysts per brain [23]. Virulence was evaluated based on the percentage of dead mice among the *T. gondii*-positive mice.

### 2.7. Statistical Analysis

Statistical analyses were performed using GraphPad Prism 8.0 software (GraphPad Software Inc., San Diego, CA, USA). Data were analyzed using the chi-squared test or Fisher’s exact test (*p* ≤ 0.05). The numerical values are expressed as the mean ± SE.

## 3. Results

### 3.1. Clinical Findings, Pathology Lesions, and T. gondii Infection in Captive Caracal

**Figure 1:**
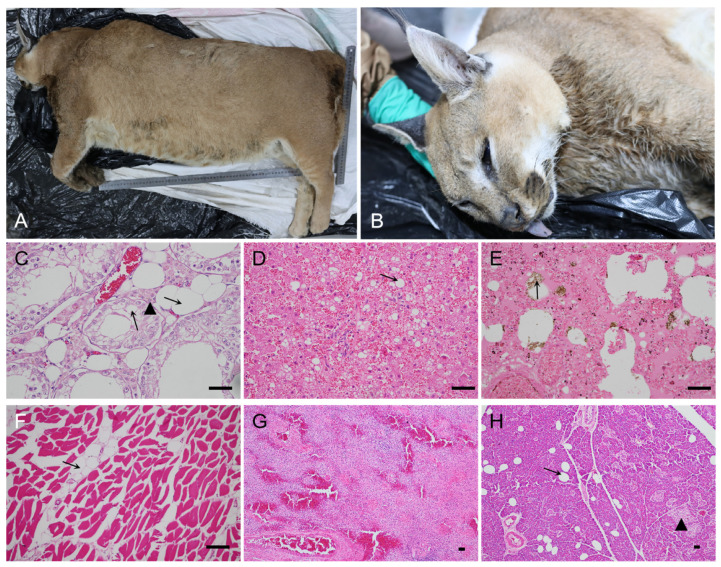
Morphology and histopathological changes in caracal case #3. (**A**): This caracal died on 8 June 2022, with a height of 54 cm, length of 76 cm, and weight of 47 kg. (**B**): The face of the caracal. (**C**): The swollen renal tubule epithelial cells with extensive cytoplasmic vacuolation (arrow), capillary congestion, brown lipofuscin granules around the cell nucleus (▲), and tubular nephropathy. (**D**): Diffuse cytoplasmic accumulation of lipids is evident (arrow), most hepatocytes are vacuolated, and nuclei have been displaced to the side; there is also hepatic lipidosis. (**E**): Transudation of protein-rich fluid (deeply eosinophilic) filling the alveoli and congested alveolar septa; there is also scattered hemosiderin deposition (arrow) and pulmonary edema. (**F**): Cardiac muscle atrophy with fatty infiltration (arrow). (**G**): Neutrophil and lymphocyte infiltration, as well as splenic trabecula with a loose structure; the vascular red pulp is markedly distended by blood, acute splenitis. (**H**): Infiltration of lipid (arrow) into the interstitium of the pancreas, necrosis (▲), and chronic pancreatitis. Scale bar = 100 μm.

One caracal from the zoo was submitted for pathological diagnosis, background, and clinical symptom assessment, and the main pathological findings of the captive caracal are summarized in Table 1. Caracal case #3, a male adult, presented with symptoms of obesity and diarrhea before death. An autopsy revealed pulmonary congestion, right ventricular hypertrophy with hydropericardium, nutmeg liver, spleen necrosis, kidney congestion, small intestinal hemorrhage, cerebrovascular rage, subcutaneous fat abundance, and joint effusion. Microscopically, the alveolar septa were widened due to congestion, edema, inflammatory cell infiltration, scattered hemosiderin deposition, hepatocyte fatty degeneration, renal tubular epithelial cell fatty degeneration, cardiac muscle and skeletal muscle atrophy with fatty infiltration, lipid infiltration, focal necrosis of the pancreas, fused ileal villi, and coagulated necrosis (Figure 1). The pathological diagnosis showed cardiac insufficiency, pulmonary edema, hepatic failure, renal insufficiency, and death due to heart failure. *Toxoplasma gondii* parasites were not observed via IHC and H&E in the tissue sections of this caracal.

*Toxoplasma gondii* IgG antibodies were found in the myocardium juice, hydropericardium, and ascites via MAT, with titers of 1:1600, 1:800, and 1:3200, respectively. *T. gondii* DNA was amplified in the tissues (heart, lungs, kidneys, tongue, diaphragm, skeletal muscle, stomach, jejunum, ileum, colon, and rectum) of the caracal with the primer pair TOX5/TOX8. The positive PCR response for *T. gondii* was strong in the intestines.

### 3.2. Isolation of Viable T. gondii from the Tissues of the Caracal Using Bioassay in Mice

Myocardial, skeletal muscle, brain, and spleen tissue homogenates from the caracal were bioassayed in Swiss mice (Tox#24-17 group) via subcutaneous injection.

**Figure 2:**
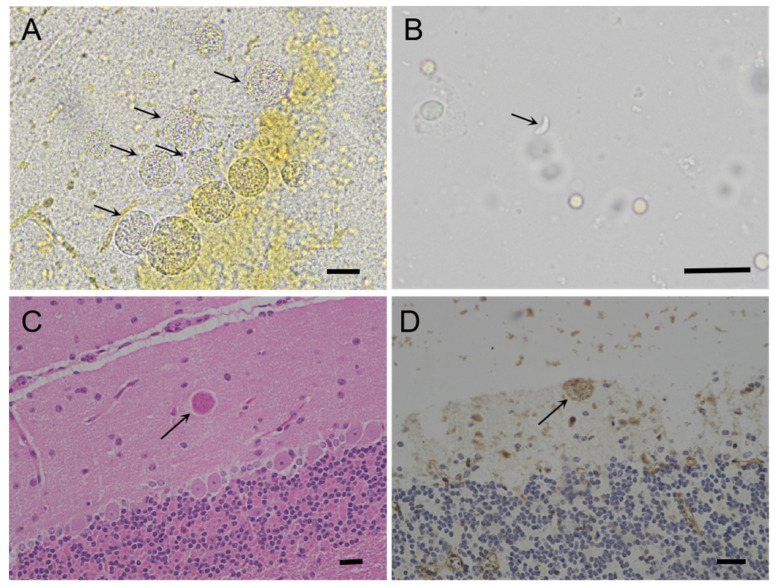
Morphology of *T. gondii* TgCaracalCHn2 in Swiss mice. (**A**): Cluster of *T. gondii* tissue cysts (arrow) in the brain of TOX 24-18 M#118, 142 DPI, unstained. (**B**): Tachyzoites (arrow) in the lungs of Tox 24-17 M#79, 27 DPI, unstained. (**C**): *Toxoplasma gondii* TgCaracalCHn2 in the brain of Tox 24-18 M#191 (arrow), 142 DPI, HE. (**D**): *Toxoplasma gondii* TgCaracalCHn2 in the brain of Tox 24-18 M#191 (arrow), 142 DPI, IHC. Bar = 50 µm.

For the Tox#24-17 group, *T. gondii* cysts were found in the brains of all mice (100%, 5/5), and tachyzoites were observed in the mice’s lungs (60%, 3/5). One of five mice (Tox#24-17, M#79) died 27 DPI with a positive titer (≥1:200) of MAT, and tissues (brain, heart, lung, and spleen) were subpassaged to Tox#24-18 and a cell culture. This isolate was successfully propagated in the cell culture (8 DPI) and designated TgCaracalCHn2. *Toxoplasma gondii* parasites were also observed in the brain of Tox#24-18 M#191 via IHC and H&E (Figure 2).

The TgCaracalCHn2-infected mice showed no obvious clinical symptoms. The main lesions in the mice were pulmonary congestion, splenic and mesenteric lymph node enlargement, and liver enlargement. Microscopically, TgCaracalCHn2-infected mice showed interstitial nephritis, adrenal gland necrosis, liver necrosis, glial tubercles, interstitial pneumonia, and acute splenitis.

### 3.3. Genotyping and Virulence of TgCaracalCHn2

**Figure 3:**
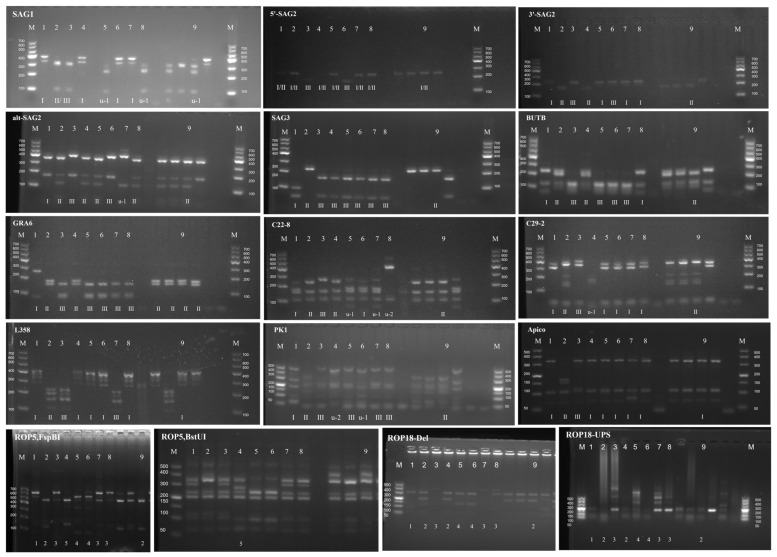
Genotyping of *T. gondii* TgCaracalCHn2 strain isolated from caracal. 1: GT1, 2: PTG, 3: CTG, 4: TgCgCal, 5: MAS, 6: TgCatBr5, 7: TgCatBr64, 8: TgToucan (TgRsCr1), 9: TgCaracalCHn2, M: markers.

DNA from the tachyzoites of TgCaracalCHn2 in a cell culture medium was analyzed using PCR-RFLP with 10 genetic markers and the polymorphic *ROP5* and *ROP18* genes. TgCaracalCHn2 was identified as ToxoDB#5 (a Type II variant). The *ROP18* and *ROP5* gene allele types of this isolate were 2/2 (Figure 3).

After injecting mice with TgCaracalCHn2 tachyzoites, the positive mice showed no symptoms within 60 DPI. In the 10^2^ tachyzoite groups, 100% (5/5) of the mice were infected with *T. gondii*. *Toxoplasma gondii* cysts (10–1010) were detected in the brains of the positive mice euthanized 60 DPI (Table 2). Compared with the other groups of tachyzoites, the number of mouse cysts in the group of 10^6^ tachyzoites was significantly increased (*p* < 0.05).

### 3.4. Morphology of TgCaracalCHn2 under Transmission Electron Microscope (TEM)

**Figure 4:**
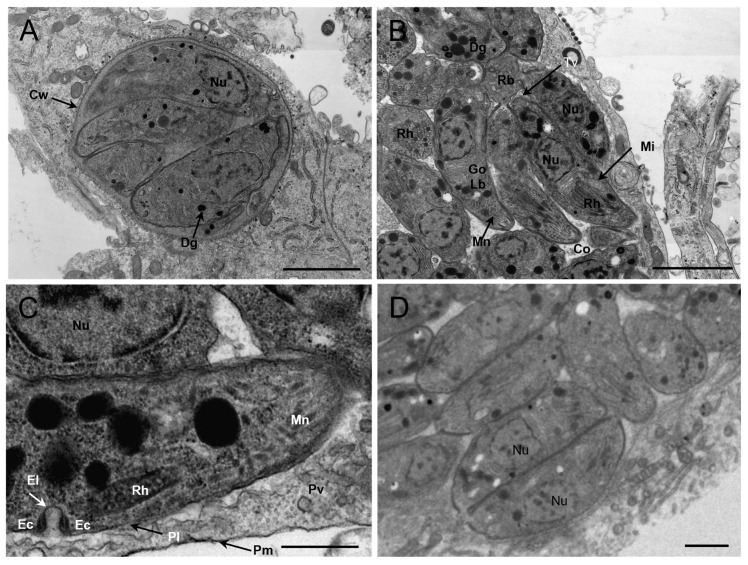
Morphology of *Toxoplasma gondii* CaracalCHn2 in cell culture (Transmission Electron Microscope). (**A**): *Toxoplasma gondii* cyst with four bradyzoites. Cyst wall (Cw: arrow), electron-dense granule (Dg: arrow). Bar = 2 µm. (**B**): A cluster of three tachyzoites in the final stages of endodyogeny still attached by their posterior ends to a common residual body (Rb); the tachyzoite contains many rhoptries (Rh) and electron-dense granules (Dg), and a lipid body (Lb). Tubulovesicular membranes (Tv: arrow), microneme (Mn: arrow), mitochondrion (Mi: arrow).Bar = 2 µm. (**C**): The parasitophorous vacuolar membrane (Pm) (black arrow) and an active micropore (Mp). Note that the micropore of the tachyzoite consists of indentation in the parasite plasmalemma (Pl) (black arrow), an electron-dense collar (Ec), and an electron-dense layer (El) (white arrow). Bar = 0.5 µm. (**D**): Tachyzoites were increased in two divisions. Bar = 1 µm. Rh: rhoptry; Nu: nucleus; Dg: electron-dense granule; Tv: Tubulovesicular membranes; Pl: plasmalemma; Pm: parasitophorous vacuolar membrane; Pv: parasitophorous vacuole; El: electron-dense layer; Ec: electron-dense collar; Mp: micropore; Go: Golgi complex; Lb: lipid body; Mn: microneme; Mi: mitochondrion; Rb: common residual body; Cw: cyst wall; Co: conoid.

A TgCaracalCHn2 cyst (size: 5.41 × 5.67 μm) was observed in the cell culture (Figure 4). The thickness of the cyst wall was uniform (0.14 ± 0.01 μm) and the outer wall film was elastic, rough, and wavy, but the inner wall was smooth. One cyst contained four bradyzoites, and the nucleus was located at the posterior end and contained abundant dense particles. Many rhoptries (3–25) and electron-dense granules (3–11) were observed in the tachyzoites. At the last stage of endosperm development, the three tachyzoites were still attached to the common residual body. Tubulovesicular membranes were also observed. The size of tachyzoites (3.67 ± 0.16 × 1.41 ± 0.06) and bradyzoites (4.75 ± 0.05 × 1.49 ± 0.17) of *T. gondii* TgCaracalCHn2 was measured using TEM.

## 4. Discussions

In this study, *T. gondii* infection was identified in a caracal from a zoo in China using serological response, nucleic acid detection, and a mouse bioassay. *Toxoplasma gondii* IgG antibodies were found in myocardium juice and edema fluid, with MAT titers above 1:800. *Toxoplasma gondii* DNA was amplified in the intestines, heart, lungs, kidney, tongue, diaphragm, skeletal muscle, and stomach. Compared with the titers of MAT in body fluids, the titer of ascites was much higher than that of myocardium juice and hydropericardium, indicating that *T. gondii* had a high load in the intestine, and this result corresponded to the PCR findings. This finding suggests that sexual reproduction may have occurred in the intestinal tissue of this caracal. The negative results for *T. gondii* oocysts or DNA in the feces of this caracal may be related to the low prevalence of *T. gondii* oocysts in field feces. According to fecal surveys, approximately 1.0% of domestic cats are expected to excrete oocysts at any given time, based on the observation that most cats excrete oocysts for about 1 week in their life [1]. Furthermore, when cats shed *T. gondii* oocysts, there is a high probability that the serum will be negative for *T. gondii* IgG antibodies.

A viable *T. gondii* strain, TgCaracalCHn2, was isolated from the tissues of this caracal, and its genotype was ToxoDB genotype #5. However, it was difficult to determine the source of *T. gondii* infection in this caracal. First, there was no inspection when the caracal was imported from Africa to China. Second, the ingestion of oocysts or bradyzoites may be the cause of *T. gondii* infection in caracal. The seroprevalence of *T. gondii* in cats from central China was 50% [24], and that in captive felids was above 80% [13,25,26,27]. This indicates that these seropositive fields have already excreted oocysts, causing widespread environmental contamination. The food of caracal includes fresh chicken, pork, beef, rodents, and insects. The seroprevalence of *T. gondii* in chickens, swine, and cattle from China was 13%, 33%, and 9%, respectively [28]. The infection rate or carry rate of *T. gondii* in rodents or insects is unknown.

TgCaracalCHn2 cysts were observed in mice’s brains. The formation of a *T. gondii* cyst depends on the host species, the genotype of the strain, the virulence of the strain, the time of the infection, the antibody titer of the host, and the route of infection [1,29,30,31]. In this study, a positive correlation was found between *T. gondii* cyst load and tachyzoite concentration. The results showed that the strain could form cysts in Vero cells, and the tachyzoites had many rhoptries and dense granules. This may be related to the capacity for cyst formation [30].

The study of the genotypes of *T. gondii* in cats and other felids is of great significance for zoonosis because they are the only hosts that can excrete oocysts and transmit this parasite to humans and other animals directly. Genotypes from domestic cats show that type II (ToxoDB genotype #1 or #3) is the predominant type in Africa, Europe, and North America, and that *Chinese 1* (ToxoDB#9, type II variation) is the most prevalent type in China; however, a diversity of genotypes was observed in South America [1,4]. This genotype distribution characteristic of *T. gondii* in domestic cats is consistent with the global *T. gondii* distribution in domestic animals and humans.

In the present study, the TgCaracalCHn2 genotype was identified as *T. gondii* ToxoDB genotype #5. ToxoDB #5 (ToxoDB #4 and #5, collectively haplotype 12, type II variation) is the predominant type (dominant type) in wild felids [1,4]. ToxoDB #5 is also the predominant type in wildlife (otters, wild swine, coyote, gray wolf, elk, moose, white-tailed deer, barn owl, bald eagle, and turkey), whereas this genotype is rare in domestic pigs, sheep, and chicken [1,14,32,33,34,35,36,37,38,39,40]. Further, ToxoDB #5 *T. gondii* is mainly identified in animals from North America but rarely in animals from the rest of the world. Here, this genotype (ToxoDB #5) was first reported outside North America. This indicates that ToxoDB #5 *T. gondii* has a sylvatic transmission cycle and a high-density pathogen load. This finding is consistent with that of Dubey et al. (2020) [34]. Little is known about ToxoDB #5 *T. gondii* infection in domestic animals and humans. However, caracals kept as pets accelerate the link between wild and domestic cycles, increase the chances of atypical genotypes emerging in domestic cats, and promote the spread of more disease-causing genotypes to humans, domestic animals, and wild animals.

In this study, the TgCaracalCHn2 strain was avirulent in Swiss mice. Few reports on clinical toxoplasmosis are associated with ToxoDB#5 of the *T. gondii* genotype in wild animals. The isolates of ToxoDB#5 *T. gondii* from sea otters (n = 117) and feral swine (n = 43) were avirulent in Swiss mice [34,41], although most sea otters died of toxoplasmosis. Furthermore, it is lethal to immunosuppressed animals and humans with toxoplasmosis.

The transmission route of *T. gondii* ToxoDB#5 in China is unknown. According to the distribution of ToxoDB#5, there are two plausible possibilities for its transmission route: (1) *T. gondii* may spread from marine animals or feral birds to land animals [42,43] and (2) *T. gondii* may spread from North America to other continents via sea trade roads [44].

## Figures and Tables

**Table 1:** Clinical symptoms and isolation of *Toxoplasma gondii* in caracal case #3 from China.

Caracal ID	Date Received	Sex, Age	Clinical Signs	Cause of Death	MAT Titers ^a^	Swiss Mouse Bioassay ^b^	*T. gondii-Positive* Tissues via PCR ^c^
Case #3 (TgCaracalCHn2)	8 June 2022	Male, Adult	Obesity, diarrhea	Cardiac insufficiency, pulmonary edema, hepatic failure, renal insufficiency.	Myocardium fluid: 1:1600 Hydropericardium: 1:800 Ascitic fluid: 1:3200.	5/5	Heart, lungs, kidney, skeletal muscles, diaphragm, tongue, stomach, jejunum, ileum, colon, rectum.

^a^: End titration. ^b^: Number of positive mice per number of inoculated mice. ^c^: Primers were TOX5 and TOX8.

**Table 2:** Evaluation of virulence of *Toxoplasma gondii* TgCaracalCHn2 strain in Swiss mice.

No. of Tachyzoites	No. of *T. gondii* Infection Mice/No. of Inoculation Mice (%)	Days of Survival/No. of *T. gondii*-Infected Mice	No. of Brain Cysts
10^6^	5/5 (100%)	≥60/5	620 ± 128
10^5^	5/5 (100%)	16/1, ≥60/4	94 ± 50
10^4^	5/5 (100%)	54/1, ≥60/4	276 ± 89
10^3^	5/5 (100%)	≥60/5	164 ± 56
10^2^	5/5 (100%)	≥60/5	86 ± 46
10^1^	2/5 (40%)	20/1, ≥60/1	162 ± 162
10^0^	1/5 (20%)	≥60/1	18 ± 18
<1	0/5 (0)	≥60/5	0
Negative control	0/5 (0)	≥60/5	0

## Data Availability

The datasets used and/or analyzed in this study are available from the corresponding author upon reasonable request.

## Abbreviations

MAT: modified agglutination test; PCR: polymerase chain reaction; H&E: hematoxylin and eosin; IHC: immunohistochemistry; TEM: transmission electron microscopy; DPI: days post-inoculation.
